# The Physiology of the Appetite

**Published:** 1910-01-29

**Authors:** E. I. Spriggs

**Affiliations:** Senior Assistant-Physician to St. George's Hospital; Physician to the Victoria Hospital for Children; Examiner in Materia Medica, Conjoint Board


### HOSPITAL CLINICS.

### THE PHYSIOLOGY OF THE APPETITE.

### WITH REFERENCE TO THE ARRANGEMENT OF MEALS AND MEDICINES.

By E. I. SPRIGGS, M.D.Lond., F.R.C.P.Lond.; Senior Assistant-Physician to St. George's Hospital; Physician to the Victoria Hospital for Children; Examiner in Materia Medica, Conjoint Board.

(Read at a meeting of the Tunbridge Wells, Eastbourne, and Hastings Divisions of the British Medical Association, held at Hastings on December 17, 1909.)

GENTLEMEN,—The possession of an appetite for food is known by all to be a sign of health. The lack of appetite is commonly the first symptom of illness to arouse the attention of a man or his friends. We do not know exactly how the need of food and water gives rise to the desire of eating and drinking, though it is probable that the sensations of hunger and thirst arise chiefly from the alimentary tract; for hunger or thirst may be satisfied at once by taking food or water into the stomach, before a sufficient time to allow of absorption or assimilation has passed. Thirst is associated with dryness of the mouth or throat, the want of secretion depending upon the concentration of the blood, to which the severity of the thirst is doubtless proportional. Hunger is associated with an empty stomach, but that is not its only cause, since it may increase in intensity after the stomach, and probably the small intestine too, is free from food.

#### PHYSIOLOGICAL APPETITE.

In a healthy man an appetite comes naturally, a few hours after a meal, upon the thought of food: if he be so occupied that no such thought enters his mind, he may be free from hunger for a much longer time. Later, however, the idea of eating will present itself, arising either from visceral sensations or from perceptions, such as the sight or smell of food, or of its preparation. In any case, as soon as the attention of a fasting man is directed to the idea of food, the appetite is aroused. If there is, at the same time, a likelihood of his obtaining food, the appetite is intensified and reinforced at once by a secretion of saliva and of gastric juice; with the result that the alimentary system is now prepared to receive and digest the food. Neither the sensations nor the secretions are excited if food has been taken recently, or if ill health has interfered with the normal process I have described.

It is necessary to obtain as full a knowledge as we can of the conditions under which the digestive juices are poured forth, in order that instructions as to hygiene, diet, and medicine may be given to those who seek advice. We have also to instruct healthy folk as to the arrangement and composition of their meals, and although it is not well for them to pay too much attention to the details of their daily food, it cannot be doubted that a neglect of all regulations is likely to predispose to digestive disorders. It is true that the human stomach would not have survived to the present day in the form in which we find it, were it not capable of great adaptation. It is possible, however, and even likely, that the evolution of the digestive organs is not now proceeding along the best lines. Civilised life, as we know it, does not tend to produce a hardy digestion. It is, therefore, our duty to inform the healthy, as well as the sick, of the circumstances which have been observed to govern a natural digestion and nutrition.

#### EXPERIMENTS IN DIGESTION.

I shall refer to-day, not so much to the teachings of experience as to the findings of experimental medicine, for by experiment alone can we determine which part of the tradition we have received is true and which false. When, as the result of a series of experiments, some fact is laid down with which we feel we were perfectly familiar before, there is sometimes a feeling of disappointment, such as I expect you will have to-day, gentlemen, for I shall lay no sensational results before you. We are apt to forget that the conclusion reached was previously but one among many other competing statements or hypotheses, whereas now it may form a firm base for further observations.

The experimental study of digestion received its first great stimulus in modern times from the observations of Beaumont[1] upon the gastric fistula of Alexis St. Martin. Beaumont's results have been confirmed and amplified recently by the researches carried out by Pawlow[12] and his pupils upon dogs. Still more recently, many of Pawlow's conclusions have been established in man.

Pawlow's dogs were provided by operation with an œsophageal fistula, the gullet being divided in the neck, and both its cut ends caused to heal separately in the wound, so that food swallowed did not reach the stomach, but passed out by the fistula. A gastric fistula was also made. Further, a part of the stomach, following Heidenhein, was separated from the rest of the organ by an ingenious operation so that an additional "small stomach" or bag of gastric mucous membrane was formed, having an external opening through the skin, and retaining all its nervous and vascular connections. By this means the secretion of gastric juice during the act of digestion in the large stomach could be watched in the small stomach free from the presence of swallowed food.

Pawlow observed that the sight of food was sufficient to cause a flow of juice in a hungry dog, and that feeding the animal with meat, which, of course, did not reach the stomach but passed out by the œsophageal fistula, known as "sham feeding," was accompanied by a copious flow of gastric juice. As much as 700 c.c., or a pint and a quarter, was obtained from a dog which ate meat for five to six hours. It is obvious, from this experiment, that the stomach can supply very large demands in health.

I do not know of any observations upon human beings directly comparable to those upon dogs with the "small stomach." There is, however, a small series of three cases of œsophageal stricture, treated by gastrostomy, in which the secretion of gastric juice has been carefully observed and reported. These reports are of great value to us in that as they confirm, on the whole, Pawlow's results, they show us that we are upon safe ground in applying them to mankind.

### Some Clinical Observations.

A. F. Hornborg[9] (1903) observed a child of five years suffering from an œsophageal stenosis due to swallowing caustic alkali, upon whom gastrostomy had been performed. The boy produced a copious secretion in the stomach if he chewed good tasting food, after a latent period of six to seven minutes. The secretion lasted forty minutes, a duration similar to that of one hour observed in dogs sham fed with meat for a short time. Chewing food which he disliked, or irritating substances, was not accompanied by secretion. The sight of food did not provoke a secretion in this boy. It is possible that when shown it he knew he was not going to have it, for even in dogs it has been found that if they believe food is exhibited for merely experimental purposes no juice flows.

H. Bogen[4] (1907) observed a similar case in a boy of three and a half years. The sight or suggestion of food was followed by a copious flow of juice after a latent period of five minutes in the case of meat and nine with milk. Any perception which he learned to associate with the idea of food, such as that of the sound of a trumpet at meal times, would arouse the secretion.

H. Katznelson[10] (1907) reported the case of a girl who at the age of fifteen acquired an œsophageal stricture through caustic alkali, was provided with a gastric fistula, and till the age of twenty-three lived entirely upon food placed in the stomach through this opening. Œsophagotomy was then done at the level of the lower part of the thyroid cartilage, the upper end of the gullet being brought out on the left side of the neck and fitted, after healing, with a tube which passed down the front of the chest to the stomach opening. At the time of Katznelson's paper this tube had been worn for eight years. The patient had put on weight and was normal in appearance, the apparatus being concealed by the clothes. The process of secretion could be observed when she lay on the left side, for if the tube was removed the gastric juice secreted ran out of the fistula. In this patient also the act of chewing and swallowing food, which passed out of the œsophageal opening, was accompanied by a copious secretion. A mixed meal of meat and vegetables called forth more secretory activity than milk. The latent period was about five minutes, and the secretion, once started, went on a long time after the sham feeding had ceased, *e.g.* in one case an hour and twenty minutes.

### Psychical Influences.

The gastric juice which is poured forth on the thought, sight, smell, or taste of food, independently of whether any food enters the stomach or not, is known as the appetite juice, and the nervous impulses which provoke its secretion are carried to the stomach by the vagus. The secretion of appetite juice is peculiarly subject to sensory and psychical influences, to which I must now refer, as this aspect of the matter is of importance to us in treating patients in whom a natural appetite is wanting.

Pain or discomfort inhibits the secretion of the juice for a long time. Indeed, the neglect of proper precautions to secure that the experimental animal was free from pain or distress led to the failure of early workers to prove the occurrence of digestive secretion or stimulation of the tenth nerve. Psychical influences of various kinds will affect the flow. I have mentioned that a dog which is led to believe, from its past experience, that food shown is not going to be given it to eat, does not produce a proper flow of appetite juice, whereas the lively expectation of a meal will do so before any food is taken into the mouth. Preoccupation of the mind and strong emotions will also inhibit the appetite juice. For instance, Bickel and Sasaki[2], after sham feeding a dog for a short time, collected 67 c.c. of gastric juice in twenty minutes. On another occasion the dog was first shown a cat, and put itself into a tremendous excitement, with the result that, under conditions otherwise similar, only 9 c.c. of juice were produced.

The child observed by Bogen, if the food which he had been led to expect was not brought, became exceedingly angry, with the effect that when his meal did appear the secretion of gastric juice was much diminished. Obvious lessons may be drawn from this experiment and applied to domestic life, but I hesitate, gentlemen, to take sides in the matter. You may have seen the cautious inscription on the obelisk near the battlefield of Naseby, stating that that great day furnishes a lesson to kings not to exceed their just prerogative, and to subjects not to rebel against their sovereign. With similar prudence, I may point out that the above experiment is a warning to wives so to order their households that dinner shall not be late, and to husbands to preserve a calm demeanour if unavoidable delays occur.

### Emotional States and Digestion.

Anger or discomfort will arrest, not only the secretion, but also the rhythmic movements of the stomach and intestines, as was observed by Canon[5] in the cat by examination with the $x$-rays after a meal containing bismuth had been given.

The evidence before us, therefore, confirms and puts upon a scientific basis the experience of mankind that pain and discomfort, preoccupation of the mind, strong emotions, and bolting the food without proper mastication are prejudicial to easy digestion. Meals should be taken in comfort among pleasant surroundings, with good temper, and at leisure, if "good digestion" is to "wait on appetite, and health on both." The personal preparation for chief meals, such as washing, changing clothes, all form part of the anticipation and predispose to a good supply of appetite juice. The sound of the dinner-bell and the clink of plates supply the final stimulus to the secretory centres. To read at meals, to converse on subjects requiring concentrated thought, or on contentious and disagreeable subjects, is undesirable. It is clear, also, from the above quoted experiment with the dog and cat, that the preference which we all have to sit at meat among friends and as far as possible from our enemies, if we have any, or, shall I say, from those with whom we have little in common, is founded upon physiological laws. It is better for us; also for them.

The food entering the stomach is collected at the cardiac end. In the centre of this mass the digestion of starch goes on for half-an-hour, by means of the saliva with which the food is intimately mixed by mastication. The gastric juice meanwhile gradually permeates the mass from without, and as each portion becomes acid salivary action is arrested, and the digestion of protein begins. The products of protein digestion themselves set up a mechanism for the continued production of juice, a chemical and local stimulus, so that the flow of gastric juice can now be continued independently of nervous impulses coming through the vagus.

We see, therefore, that unless the appetite juice be first forthcoming to start the digestion of protein, the stimulus for the second or chemical flow will be wanting. This has been shown experimentally both in dogs and in man. In the former, if meat be placed in the stomach without the knowledge of the animal it may lie there undigested for hours, and may even decompose without giving rise to a flow of gastric juice. In the latter, Schüle[15] recorded that, in a patient whose stomach was being washed out daily, if food was introduced through the tube without the knowledge of the patient, so that the thought of food which would arouse the appetite juice was wanting, then on withdrawing the stomach contents later it was found that but little digestion had taken place.

### Aids to Digestion.

It follows that the loss of appetite bears a double penalty, first the loss of the appetite juice, and secondly, the loss of the chemical secretion of juice. The latter, however, can be aroused if the products of protein digestion, artificially prepared, are placed in the stomach. These are contained in soups made by stewing meat or bones. Soup is, therefore, as is well known, a help to digestion if the appetite is poor, and should be taken, as it is, near the beginning of a meal; it is neither necessary nor advisable to take a great deal of it.

The pancreatic secretion is, as shown by Bayliss and Starling, excited by a hormone or chemical messenger which is formed when the first acid chyme from the stomach reaches the duodenum, and is carried by the blood to the pancreas. At the same time and in the same way a flow of bile and intestinal juice is called forth. We have but little reliable knowledge of defects of these juices, apart from gross obstruction of ducts or severe inflammation of the glands producing them.

We may now look at the information we have as to the effect of different kinds of food upon secretion, and consider what further guidance is given us.

The amount of salivary, gastric and pancreatic juice poured forth increases with the amount of food, but it is not the same at different times, especially as regards the appetite juice, which, as we have seen, varies with so many circumstances. The supply of digestive juices in health is plentiful and difficult to exhaust.

Meat, alone or mixed with bread, calls forth the greatest flow of gastric juice, and the secretion lasts longer than is the case after a meal of starchy foods. A meat meal remains in the stomach much longer than one of carbohydrate or fat.[6] In the dog the secretion of gastric juice after a meal of meat lasts four hours, and that of pancreatic juice five hours. The gastric flow reaches its maximum in one hour or a little over.

### Foods and Digestibility.

It is obvious that the digestion of food, involving so much secretory, motor, and absorptive activity, causes a good deal of energy to be used up. Accordingly it is found that there is a considerable increase of oxydation, as measured by the respiratory exchange, after a meal. Further, the increased oxydation is 30 per cent. greater after a meal of meat than after one of carbohydrate and fat, and lasts four hours. From the two sets of data, namely, the time of continued secretion and the time of increased oxydation, we see that four or five hours elapse before the bodily activities aroused by a meal of protein are at rest again. We may, therefore, conclude that in health a meal of meat should not be followed by another meal for at least four to five hours. If it be necessary to give food after a shorter interval, it should be of a non-protein character. Further, since the maximum secretion of juice after meat is not reached for an hour, it is reasonable to recommend that, if possible, an hour's rest, or at least freedom from active or anxious employment, should follow the chief meal of the day.

Egg-white leaves the stomach with little delay and is, therefore, a suitable protein to use when meat cannot be allowed.

Bread calls forth a good flow of gastric juice, but is passed out of the stomach fairly quickly, as are carbohydrates generally. Hence, small meals taken between larger ones should consist chiefly of carbohydrate. Biscuits, bread, butter, and cake are used for this purpose. Whenever the digestion is ailing, or when meals are being taken near together, foods containing chiefly carbohydrate should be as dry as possible, such as dry or stale bread, toast, and biscuits, which require to be well masticated and are, therefore, thoroughly mixed with the saliva before they are swallowed, and afterwards with the other digestive juices. Dry toast buttered is more easily digested than hot buttered toast, which is soaked through with fat.

Fats inhibit the gastric juice and, though they do not stay in the stomach so long as proteins[6] are only passed gradually into the duodenum. Fat should, therefore, not be given in large quantities, and not in small intercurrent meals, but in the larger meals. The comparatively small amount of fat in one or two pieces of bread and butter probably does not have much influence; indeed, butyric acid has an opposite effect, it is a stimulant of the gastric juice. Perhaps this is a reason why butter is borne so much better than other fats.

Milk contains all the three foodstuffs, but it is digested with less secretory activity than any other food, and is, therefore, most suitable for the uses to which it is put in disease. The secretion of gastric juice is not great, firstly because there is but little appetite juice, and secondly because the fat in the milk has its inhibitory effect. Neither is much pancreatic juice called forth. Cream hinders gastric secretion still more strongly. Lactic acid, however, exerts a favourable influence, though not so favourable as that of butyric acid.

Exclusive diets should be avoided. A true exclusive diet consisting of only one of the three foodstuffs cannot be endured by the body for long. A mixture of food is required to sustain the tissues and functions in health. The study of the appetite shows us that the appetite and the juice it calls forth are more easily excited by a pleasing variety of food, and a diet of this nature is, therefore, indicated. Prolonged meals consisting of a large number of dishes, however, act in the opposite direction and if repeated often are harmful to the digestion. Two or three dishes, well cooked, should be sufficient for all.

### Digestive Stimulants.

If the appetite is jaded, the eating of highly-tasting foods, as hors d'œuvres, will stimulate the appetite juice, whilst soup, as we have seen, will call forth the second or chemical flow of juice. In perfect health, such preliminaries to a meal should not be necessary, unless indeed, as is doubtless sometimes the case, it is desired that the appetite be aroused to an unusual degree and the moderate needs of nutrition subordinated to the pleasures of the palate. Savouries, again, taken towards the end of a meal, are not necessary for digestion, though pleasant in themselves.

We have seen that the products of the digestion of protein are the most efficient arousers of the second flow of juice, also that protein and fat take longer to be discharged from the stomach than carbohydrate. It follows that in a mixed meal the protein and fat should be taken early in the meal, the carbohydrate later.

I have mentioned above that in a thirsty person the secretions generally are suspended. Pawlow showed that a good supply of fluid was necessary for the production of digestive juices. An observation of Bickel's[3] illustrates this. A dog which had taken no food or water for sixteen hours produced very little juice on sham feeding. Three hundred c.c. of water were then put into the stomach. After ten minutes 250 c.c. of this had been passed on or absorbed. The remaining 50 c.c. were removed and the animal again allowed to eat meat. A plentiful secretion followed, lasting for one hour.

### Meal Times.

We are often asked at what time to take fluid, before, during, or after a meal? The answer has not, in the past, been easy to give because of the fear of diluting the digestive juices, which may be none too plentiful or active. Experiment has shown that if water is given before a meal it is passed out of the stomach in from ten to twenty-five minutes. It now appears that when a fair quantity of fluid is taken in the course of a meal consisting chiefly of solids it is separated from the solids, which lie in a mass in the cardiac end of the stomach, and is also quickly discharged from the duodenum, sometimes with barely an acid reaction. In the light of these observations, it does not appear to be a matter of consequence whether fluid in health is taken before, during, or immediately after a meal. It is not likely to dilute the gastric contents, unless perhaps it is continually sipped between mouthfuls of food. Water passed into the duodenum is soon absorbed. It is not probable that any harm may result from diluting the intestinal contents by large draughts of water, for in experiments in which enormous quantities have been taken daily, the absorption of food, as ascertained by analyses of the fæces, has been found to be normal.[7]

### Fluids.

In most forms of dyspepsia, dilated stomach, obesity, and other conditions in which it is desirable that only small meals should be taken, it is of advantage to advise that no fluid be drunk until the meal is over. Drinking fluid during a meal restores the appetite to some extent and enables more food to be eaten. Water is an excitant of gastric juice.

Ice-cold water, on the contrary, is a powerful depressant of secretion, not only where it is directly applied experimentally in the stomach, but in the small stomach as well. It is known that the constant drinking of ice water is harmful to the digestion. Ices ought to be refused by those of poor digestion, and by the healthy should be taken into the mouth in small quantities only and allowed to be completely melted and warmed before swallowing.

Fluids are needed in greater quantity when much meat is eaten, whilst those taking a low protein diet need much less water and pass out less urine.

The time at which the chief meals of the day are taken differs among different races. We have seen reason above to lay down as a rule that a meal containing much protein should be followed by an interval of four or five hours. It is also clear that chief meals should be eaten under conditions of freedom from the thoughts of work. The solid breakfast, an ancient British institution, is taken after the night's rest when the body generally is of good tone; the knee-jerk, for instance, is brisker in the morning. I do not doubt that the digestive organs are also in better condition at this time, for foods which later in the day are ill-borne in dyspeptic subjects, such as bacon, can often be relished at breakfast without penalty. The desire to rest idle after a good meal is also frequently absent at this hour, though present after later meals. In the morning the digestive system is more like that of a child, who seldom desires to rest after any meal. A meat breakfast can, therefore, be recommended, provided that time be allowed to eat it at leisure.

### Number of Meals.

Not more than two meat meals need be advised in the day to healthy people. The question of whether the second meal should be taken at midday or in the evening must be decided after consideration of the claims of work. If breakfast be early, say eight o'clock, and at midday an hour and a half or two hours to eat and rest be allowed, as in many parts of the Continent, there can be little doubt that to take the chief meal of the day between one and two is a good habit. The working day is divided into two parts, and the occupation can be carried well into the evening without undue fatigue. The necessities of modern life, however, render this plan difficult to the great mass of workers in towns who are far from their homes and can at the most give an hour, and often less, to their midday refreshment. Under these conditions, the midday meal should be light, containing but little meat, with carbohydrate and fruit, and no alcohol. The chief meal will then be taken in the evening, when the mind will be, or should be, relieved of the anxieties of the day. But if the work has been strenuous, the man may be tired in the evening, and his digestion not in the best state to do its work. In such a case, great benefit is derived from half an hour's complete rest in an easy chair, or lying down, before the evening meal, a plan found of great value in sanatoria, where the nutrition of the patient is the chief aim.

Intercurrent meals, such as mid-morning lunch and afternoon tea, should be small and consist almost entirely of carbohydrate, so that the stomach may be empty before the meat meal. Protein foods, such as sandwiches of meat and potted meat, should be avoided.

### Tea Drinking.

Tea, if freshly made and poured off the leaves, has an aroma and taste which stimulate the appetite; the caffeine it contains has, when taken in moderation, an excellent effect upon the central nervous system, and restores the flagging muscles. The ingestion of warm fluid is also refreshing. We know, however, that strong tea is the cause of dyspepsia, and directly inhibits the secretion of gastric juice. In Sasaki's experiments[14] the effect of sham feeding in a dog operated upon by the Pawlow method was noted when no tea had been taken and when a strong infusion, made by putting ten parts of tea to 400 of boiling water, had been placed in the stomach for fifteen minutes. In the latter case the flow of gastric juice was much smaller.

Late suppers are well known to disturb sleep if the digestion be not perfect. The secretion of pancreatic juice goes on during sleep when food is to be digested,[12] and so do the movements of the small intestine.[5] Sleep is, therefore, as is to be expected, no bar to digestion. We must reflect, however, that if the evening meal be taken early, say between seven and eight o'clock, the food will be digested before midnight and the digestive system will have a long rest before the morning meal—will have, in fact, its night's repose; whereas, if a supper be taken immediately before retiring, the stomach and intestines will not get to their rest until the early hours of the morning.

### Dyspepsia and its Treatment.

I may now mention a few points raised by the experimental work I have been discussing, which bear upon the treatment of sufferers from the various forms of dyspepsia associated with loss of appetite. Pawlow observed that a dog which was the subject of a gastric catarrh produced an incessant slimy acid secretion. In such an animal a flow of appetite juice might be obtained, but the second chemical secretion was not. Two lines of treatment are suggested, one, to arrest the incessant flow of juice periodically so as to give the glands an opportunity to recover, and the other to arrange the food so that the appetite juice alone may be expected to digest it. To effect the latter, only small meals should be given, and these at more frequent intervals than in health. For the former we may make use of bicarbonate of soda.

Bicarbonate of soda, if given in suitable quantity, inhibits the secretion without, so far as we know, harming the glands, and, therefore, gives them rest. A solution of a strength of a quarter to a half per cent., according to Lönnquist,[11] has no influence beyond the stimulating one of the water in which it is dissolved. A solution of one to one and a half per cent. definitely lessens the secretion. One and a half per cent. is about seven grains to the ounce. We should, therefore, remembering that the medicine will be diluted by the stomach contents, give at least twenty grains in a dose. The effect reaches its maximum at the end of the first hour or the beginning of the second. It appears, therefore, that when bicarbonate of soda is being prescribed with this object it should be taken about an hour and a half after food. It will not then interfere with the digestion of the small meal preceding it, whilst its action will have passed off by the time the next meal is taken, supposing that a meal is being given every three hours. Magnesia has a much slighter effect.[8]

In hyperæsthetic conditions of the stomach, soda gives great benefit by neutralising the acidity of the juice, and in these cases it may be necessary to administer it earlier after a meal, though in very many it will be found that a regular dose an hour and a half after food will soon bring relief. The mucus poured out in gastric catarrh reduces the efficiency of the gastric juice by combining with its acid. Any strong irritants, such as acids or concentrated salts, increase the secretion of mucus enormously, even, according to Pawlow, a hundredfold. In diseased states, therefore, any form of irritating or peppery food should be forbidden.

### Bitters and Peptic Ferments.

Bitters, when given immediately before food, increase the secretion of the appetite juice. The effect is apparently due to the contrast between their taste and the taste of food. From observations on dogs it is possible that the same result would be obtained if the mouth were well washed out with the bitter solution. Such solutions given immediately before meals, should, in accordance with several time-honoured formulæ, be combined with acids, rather than alkalis. The recent observations of Heinsheimer[8] on the influence of drugs upon secretion showed that bismuth subnitrate diminishes secretion to some extent, but has no effect on the digestion of protein or the acidity of the juice.

The preparations of the peptic and pancreatic ferments are now put forward as helps to digestion. When the gastric juice is deficient, however, the fault lies usually in the non-secretion of acid. Lönnquist[11] and Rosenberg[13] have recently investigated this question. The former found in the dog that neither .1 to .5 per cent. hydrochloric acid nor the natural juice called forth much secretion. Nevertheless, acid when added to juice deficient in ferment increased its activity. Rosenberg administered fresh, active, human gastric juice to patients with achlorhydria, achylia, and chronic gastritis with subacidity, in doses of one to four drachms four times a day ten minutes before food. The stomach washings were analysed weekly. No definite alteration in the proteolytic power or the acidity of the gastric juice or in the motor power of the stomach was detected. In one of the patients much undigested connective tissue was found in the fæces, and was still present after the treatment. Some patients, however, expressed the opinion that the appetite and subjective symptoms were improved. Rosenberg concludes that no better results were obtained from giving gastric juice artificially than from prescribing a corresponding quantity of dilute hydrochloric acid. If this is true of fresh, active juice, we should not expect dried preparations to be of greater, or, indeed, of equal, value. There is but little accurate experimental work to guide us in the administration of pancreatic ferments in cases of supposed intestinal dyspepsia, not due to gross lesions of the mucous membrane or of the glands.

Finally, gentlemen, I may remark that, whilst we may follow with confidence the paths marked out for us by careful research, we must not forget that in medicine no rules are absolute; and, although I have quoted them in a former lecture, I may recall to your minds Bacon's words: "Physicians are some of them so pleasing and conformable to the humour of the patient, as they press not the true cure of the disease; and some other are so regular in proceeding according to art for the disease, as they respect not sufficiently the condition of the patient. Take one of a middle temper; or if it may not be found in one man, combine two of either sort; and forget not to call as well the best acquainted with your body, as the best reputed of for his faculty."

1 Beaumont, Exps. and obs. on gastric juice, 1834.
2 Bickel, A., and Sasaki, T., Deutsch. med. Wochenschr., 1905, xxxi., 1829.
3 Bickel, A., see No. 10.
4 Bogen, H., Arch. f. d. ges. Physiol., 1907, cxvii., 150.
5 Cannon, Amer. Journ. of Physiol., 1902, vi., 251.
6 Cannon, Proc. Amer. Physiol. Soc. *Ibid.*, 1903, viii., xxii.
7 Gruzdiev, A Digest of Metabolism Experiments. Atwater and Langworthy, Washington, 1898, p. 188.
8 Heinsheimer, F., Med. Klinik., 1906, p. 616.
9 Hornborg, A. F. Münch. med. Wochenschr., 1903, l., 1308.
10 Katznelson, H., Arch. f. d. ges. Physiol., 1907, cxviii., 327.
11 Lönnquist, B., Skand. Arch. f. Physiol., 1906, xviii. 194.
12 Pawlow, J.P., The Work of the Digestive Glands. Trans. by W. H. Thompson. London, 1902.
13 Rosenberg, E., Münch. med. Wochenschr., 1907, liv., 1272.
14 Sasaki, T., Berl. klin. Wochenschr., 1905, No. 49.
15 Schüle, Deutsch. Arch. f. klin. Med., 1901, lxxi., 111.

